# The Atlantic Salmon Gill Transcriptome Response in a Natural Outbreak of Salmon Gill Pox Virus Infection Reveals New Biomarkers of Gill Pathology and Suppression of Mucosal Defense

**DOI:** 10.3389/fimmu.2020.02154

**Published:** 2020-09-04

**Authors:** Mona C. Gjessing, Aleksei Krasnov, Gerrit Timmerhaus, Svante Brun, Sergey Afanasyev, Ole Bendik Dale, Maria K. Dahle

**Affiliations:** ^1^Department of Fish Health, Norwegian Veterinary Institute, Oslo, Norway; ^2^Norwegian Institute of Food, Fisheries and Aquaculture Research, Tromsø, Norway; ^3^MOWI ASA, Bergen, Norway; ^4^Sechenov Institute of Evolutionary Physiology and Biochemistry, Saint Petersburg, Russia; ^5^The Norwegian College of Fishery Science, UiT – The Arctic University of Norway, Tromsø, Norway

**Keywords:** salmon gill poxvirus, Atlantic salmon, gill disease, transcriptome, aquaculture, smoltification

## Abstract

The salmon gill poxvirus (SGPV) is a large DNA virus that infects gill epithelial cells in Atlantic salmon and is associated with acute high mortality disease outbreaks in aquaculture. The pathological effects of SGPV infection include gill epithelial apoptosis in the acute phase of the disease and hyperplasia of gill epithelial cells in surviving fish, causing damage to the gill respiratory surface. In this study, we sampled gills from Atlantic salmon presmolts during a natural outbreak of SGPV disease (SGPVD). Samples covered the early phase of infection, the acute mortality phase, the resolving phase of the disease and control fish from the same group and facility. Mortality, the presence and level of SGPV and gill epithelial apoptosis were clearly associated. The gene expression pattern in the acute phase of SGPVD was in tune with the pathological findings and revealed novel transcript-based disease biomarkers, including pro-apoptotic and proliferative genes, along with changes in expression of ion channels and mucins. The innate antiviral response was strongly upregulated in infected gills and chemokine expression was altered. The regenerating phase did not reveal adaptive immune activity within the study period, but several immune effector genes involved in mucosal protection were downregulated into the late phase, indicating that SGPV infection could compromise mucosal defense. These data provide novel insight into the infection mechanisms and host interaction of SGPV.

## Introduction

Atlantic salmon (*Salmo salar* L.) are mainly farmed in cold waters in Northern Europe, North America, Chile and Tasmania. The anadromous salmon life cycle is mimicked in aquaculture, where juvenile fish are farmed in indoor fresh water facilities, artificially smoltified, and then transferred to sea water for the grow-out phase. The Norwegian coastline currently represents the main Atlantic salmon production area. Infectious diseases are challenging at all phases of the salmon production cycle. Several pathogenic microorganisms, including amoeba, bacteria and viruses, target the gill and can cause destruction of the gill epithelial surface, harm respiratory function and lead to mortality, compromised animal welfare and big economical losses (1).

An Atlantic salmon gill disease leading to high mortalities in presmolts, characterized by respiratory distress and severe gill pathology, was observed in salmon aquaculture from 1995 (2). The disease was suspected to be caused by a virus (2), and in 2015 the salmon gill poxvirus (SGPV) genome was sequenced from diseased gills and partly characterized (3). The disease was then named SGPV disease (SGPVD) and is most easily recognized when it is manifested with sudden high, acute mortality and severe gill lesions, typically occurring in juveniles in presmolt facilities (3), but also described in fry (4), and wild salmon (5). Typical microscopic observations in the gills are apoptotic gill epithelial cells and sometimes changes in the chloride cells in the gills in the acute phase, with hyperplastic changes in the gill epithelium in salmon that have recovered from a SGPVD outbreak (3, 6). These observations correspond to the clinical manifestation of fish in severe respiratory distress, since the lesions lead to significant reduction of the gill respiratory surface. SGPV is shown to be spread by horizontal transmission through water (7), and was recently shown to reproduce SGPVD in an experimental trial (8).

Poxviruses are large enveloped viruses (approx. 350 nm), with a complex morphology and a linear, double stranded DNA genome which can contain more than 200 genes (3, 9). Poxviruses replicate entirely in the cell cytoplasm using their own polymerases rather than entering the cell nucleus (10). In the virus particle, the genome is associated with nucleoprotein and encapsulated in a protein core (9). When spreading, the viruses bud through the plasma membrane, and aquire a protein-containing membrane (11). A single membrane covered poxvirus is primarily involved in transmission between hosts, whereas additional membranes can be added to the virus when it first buds into the golgi apparatus before leaving the cell, and the double membrane virion is shown to be mainly involved in cell-cell dissemination within a single host (11). The transcription of viral genes is divided into early, intermediate and late transcription, through a sequential expression of transcription factors from the genome (12). After entry of the virus core into the cell, early gene expression produce proteins needed for DNA synthesis and for the onset of intermediate and finally late genes, which encode viral assembly proteins (13). Genes that are centrally located in the poxvirus genome are most conserved, and primarily required for replication and virus particle components, whereas a more variable set of genes are terminally located. Such genes tend to be involved in host range restriction and to restrain the immune system (10). Most of the chordopoxviruses characterized so far have distinct and comparable gene orders. In contrast, the gene order for SGPV is different from the chordopoxviruses (3). About a third of the genes encoded by SGPV have no obvious homology to other poxviruses, and their functions are completely unknown.

The host response to viral infection includes a wide range of antiviral effector mechanisms which can limit viral production, and thereby disease outcome and further viral spread. The antiviral response is counteracted by viral host-interaction mechanisms (14). Whereas many smaller viruses rely on a few proteins involved in counteraction of host defense, large DNA-viruses have developed a very complex system of communication with the host, inhibiting both intracellular and extracellular antiviral responses (14). The understanding of host-SGPV interaction is limited, and so are disease mechanisms and host responses to the virus.

The mucosal defense system in gills consists of several innate and adaptive immune mechanisms to protect the tissue from viral infection (15). Upon infection, the composition of the mucus alters, proteases and antiviral peptides are activated, and the production of cytokines and chemokines are induced to recruit and activate immune cells, including mucosal “innate” T-cells and IgT + B cells. When viral antigens are presented by infected cells and the right cytokines and chemokine environment induced, IgT + B cells can proliferate and generate pathogen-specific IgT locally in gills (16), leading to neutralization of viruses.

In presmolt facilities, SGPVD is reported to have a distinct manifestation with high, acute mortalities and with close correlation between SGPV levels, pathology and mortality (6). The virus can also be present at low levels in fish without clinical disease. Genetic variants of SGPV have been explored using full genome sequencing and multiple locus variable number of tandem repeats (VNTR) assay (MLVA) (17), but no clear link between genetic variants, and disease has been reported so far. However, according to experiences from field outbreak of SGPVD and to experimental trials with hydrocortisone treatment, the onset of clinical SGPVD has been linked to stress (8). The gill diseases in the sea water phase are often more complex with multifactorial etiology (6), and hard to interpret. Based on diagnostic experiences, SGPV is belived to operate as a primary pathogen disrupting the epithelial barrier paving the way for secondary infections (6). However, this is so far based on a set of clinical and pathological observations, and the long term effects of the SGPV infection are still unknown.

Here, we aimed to characterize Atlantic salmon gill responses in an acute high mortality SGPVD outbreak in a presmolt facility with juvenile fish, which based on clinical, histological and viral investigations was identified as a clear cut and typical case of SGPVD. The purpose of the study was to increase the understanding of the virus-host interaction mechanisms in the SGPV-infected gills, and potentially identify some specific response patterns that may have future biomarker potential in order to predict the outcome of an SGPV infection. We explored the gill transcriptome response to infection using an oligonucleotide microarray, studying samples representative for the early pre-mortality phase, the peak mortality/pathology phase, and the late regenerating phase of the disease.

## Materials and Methods

### Clinical Observations and Sampling

Samples were collected during a characteristic SGPVD outbreak in a Norwegian salmon hatchery with 57 tanks and flow through water supply, producing 8,3 mill smolts per year. To cover different stages of the disease course, fish were sampled from tanks with different disease status, and at three different time points during the outbreak. Gills, spleen and kidney were sampled on neutral phosphate buffered 10% formalin, and gill samples were collected on RNALater (QIAGEN). At the first sampling, May 10th 2017, fourteen clinically diseased fish with an average weight of 28 grams (Tank I) were sampled at the initial day of the disease along with ten fish of the same size from a clinically unaffected tank (Tank III). The second sampling was performed after 8 days, when the mortality had ceased in tank I, and eight of the remaining fish were sampled (Table 1). 5 days later (3rd sampling), eight fish with an average weight of 32 grams were sampled from a new tank (tank II), where ongoing disease had been observed for 2 days (Table 1). Eight seemingly healthy fish were sampled from tank III at the same time point. Their average weight were then 41 grams. 10 days later, a characteristic SGPVD outbreak also occurred in tank III.

**TABLE 1:** A summary of observations and analyses from the groups of the SGPV outbreak.

Date of sampling	Tank ID	Clinical state	# Fish sampled	Group ID	SGPV Pos/total	SGPV Ct median (range)	Apoptosis
10.05.17	I	Diseased	14	M-I	14/14	17,2(15,15−18,36)	14/14 extensive
18.05.17	I	Recovered	8	L-I	8/8	24,4(22,18−30,05)	2/8 sparse
23.05.17	II	Diseased	8	M-II	8/8	18,9(17,48−20,19)	8/8 Mod/extensive
10.05.17	III	Healthy	10	C-III	1/10	40(40−32,49)	0/10
23.05.17	III	Healthy	8	E-III	8/8	31,5(29,45−34,46)	0/8

### Histology and Immunohistochemistry

According to standard protocols, 3 μm-thick sections were stained with hematoxylin and eosin (H&E) for histology. A subset of gill samples were stained to detect osmoregulatory chloride cells using, an antibody directed to a conserved region of Na+/K+ −ATPase a-subunit. This antibody, a5, was obtained from the Developmental Studies Hybridoma Bank that has been widely used in studies of teleost chloride cells (4, 18). Briefly, sections were dewaxed, rehydrated, autoclaved for 15 min in citrate buffer (0.01 m, pH 6.0) at 120°C for antigen demasking, washed in distilled water, incubated for 20 min in Tris–buffered saline (TBS 0.05 m, pH 7.6) with 2.5% bovine serum albumin (BSA) for prevention of non-specific binding, tilted to remove solution, incubated for 60 min with primary antibody described above, diluted 1:100 in TBS with 2.5% BSA. A biotinylated rabbit anti mouse and alkaline phosphatase conjugated streptavidin system was used to visualize the binding.

The degree of gill epithelial apoptosis and hyperplasia and hemophagocytosis in haemopoetic organs was scored semi quantitatively from 0 to 3. For gill epithelial apoptosis, a score of 0 suggested no apoptosis,0,5- 1 sparse, where just one or a very few lamellae were affected with few apoptotic cells, 1,5- 2 suggested moderate apoptosis and 2,5-3 indicating extensive degree of apoptosis. Score 3 suggests that respiratory units without many apoptotic gill epithelial cells are difficult to find. Hemophagocytosis in haemopoetic organs was also scored from 0 to 3 suggesting no, few, moderate or extensive hemophagocytosis, respectively. Examples of scores are given in Figure 2.

### qPCR for SGPV Detection

Gill samples fixed in RNAlater^®^ (Qiagen) were homogenized in Buffer RLT (Qiagen) and DNA was isolated using the Viral DNA large Volume kit (Pathogen Universal). The quantification and purity of DNA was measured using a NanoDrop 8000 device (NanoDrop technologies). A qPCR assay directed against the SGPV D13L genomic sequence was run using a Stratagene system (Agilent) and related software (MxPro-Mx3005P), as previously described (6). Virus-specific primers and probe were employed with the following PCR parameters: 50°C for 2 min (UDG incubation), 95°C for 15 min (UDG inactivation), and 45 cycles of 94°C/15 s, 55°C/30 s, and 72°C/15 s were used. Cycle threshold (Ct) values ≥ 40 were considered negative.

### RNA Preparation for Microarray

Gill samples stored on RNALater (approx. 20 mg tissue) were transferred to 0.5 ml of Qiazol lysis reagent (Qiagen, Germany) and homogenized in a TissueLyser II (Qiagen) using 5 mm steel beads (Qiagen). Chloroform (0.1 ml) was mixed into the homogenate followed by centrifugation. The top aquatic phase was collected, mixed with 70% ethanol (1:1), and transferred to an RNeasy filter tube for isolation of total RNA according to the RNeasy kit protocol (Qiagen). Total RNA was eluated in 50 μl RNase free water, RNA concentration and purity was measured in a NanoDrop 8000 spectrophotometer, and the sample was immediately stored at −80°C until microarray analyses.

### Microarray Analysis

The transcriptome analyses on gill RNA were carried out using NOFIMA’s Atlantic salmon oligonucleotide microarray Salgeno-2 (GPL28080) and bioinformatic package STARS (19). The platform includes 44 k unique probes to protein encoding salmon transcripts. Atlantic salmon genes were annotated by functions (GO), pathways (KEGG), and custom vocabulary. Microarrays were manufactured by Agilent Technologies (Santa Clara, CA, United States) and unless indicated otherwise, the reagents and equipment were purchased from the same source. The microarray analyses were performed on 4 representative gill RNA samples from each of the five sampling groups. RNA amplification and labeling were performed with a One-Color Quick Amp Labeling Kit and a Gene Expression Hybridization kit was used for fragmentation of labeled RNA. Total RNA input for each reaction was 500 ng. After overnight hybridization in an oven (17 h, 65°C, rotation speed 0.01 *g*), arrays were washed with Gene Expression Wash Buffers and scanned. Global normalization was performed by equalizing the mean intensities of all microarrays. Next, the individual values for each feature were divided by the mean value of all samples producing expression ratios (ER). The log2-ER were calculated and normalized with the locally weighted non-linear regression (Lowess). The control samples (C-III) were used as reference. Differentially expressed genes (DEG) were selected by criteria: ER > 1.75-fold and *p* < 0.05. Enrichment of functional categories GO and KEGG pathways was assessed comparing numbers of genes among DEG and on the platform (Yates’ corrected chi-square, *p* < 0.05). Functional groups with co-ordinated expression changes were found by deviation of mean log_2_-ER from zero (*p* < 0.05, *t* test). Data were submitted to NCBI Gene Expression Omnibus (GSE151463).

## Results

### Mortality and Clinical Signs of the SGPVD Outbreak

**FIGURE 1:**
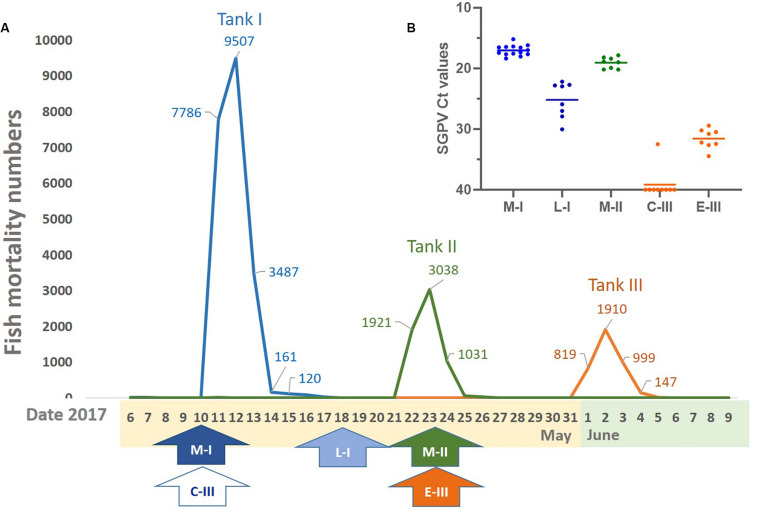
Mortality, sampling times, and virus levels. **(A)** Overview of the mortality and timeframe of the SGPV outbreak in three fish tanks. **(B)** SGPV levels analyzed by qPCR and given as mean and individual Ct values (inverted *y*-axis). Line and arrow colors: Orange (C-III, control and E-III, early infection), blue (M-I, peak infection/clinical disease and L-I, late infection/resolving disease), and green (M-II, peak infection/clinical disease).

The SGPVD outbreak occurred a few days after sorting in the spring 2017 and lasted from may 10th to june 4th with more than 200 000 Atlantic salmon presmolts dying with clinical signs typical of SGPV infection. The cumulative mortality during the outbreak was 6.6%. Of the 57 tanks in the hatchery, 40 tanks were affected, and had an increased mortality lasting for 4–18 days with a loss ranging from 310–23223 fish. The disease outbreak was characterized by loss of appetite and lethargic individuals in severe respiratory distress. In each of the tanks with fish experiencing SGPVD, mortality was observed for exactly 4 days (Figure 1A).

Fish in tank I had been sorted 4 days before the outbreak. From this tank, samples from 14 diseased fish (M-I group) were taken upon the first notice of clinical disease, which coincided with the first day of increasing mortality. Mortality peaked abruptly on the following day when about 8000 fish were lost (Figure 1A). The total mortality during the 4 days was more than 20 000 fish in this tank (Figure 1A). Then, the mortality dropped significantly, and when tank I was sampled again 8 days after the onset of disease, there were no mortality or clinical signs of disease. At this point, eight clinically healthy fish, denoted L-1 group, were sampled to monitor the late regenerating phase of disease.

Eleven days after the onset of disease in tank I, diseased fish were observed in tank II.

Fish in tank II had been sorted 13 days before the outbreak in this tank. Here, samples from 8 clinically diseased fish, denoted M-II group, were taken 2 days after the onset of disease and at the peak of mortality. Also in this tank, the high mortality lasted for 4 days leading to a total loss of about 6000 fish (Figure 1A).

Tank III was used as a control tank in the experiment, and sampled simultaneously as the outbreaks in tank I and II. The fish appeared healthy at the time of both samplings from this tank. However, a low daily loss (1–7 fish) was reported in the days after the last sampling, followed by acute high mortality after 8 days (Figure 1A) and 30 days after sorting. In tank III, the mortality peak also lasted for 4 days with a total loss of about 4000 fish (Figure 1A). The first sampling from tank III was used as negative control/reference material and denoted C-III group, whereas gills from the second sampling were found to be SGPV positive and considered early pre-outbreak samples (denoted E-III group).

### Histopathological Findings

**FIGURE 2:**
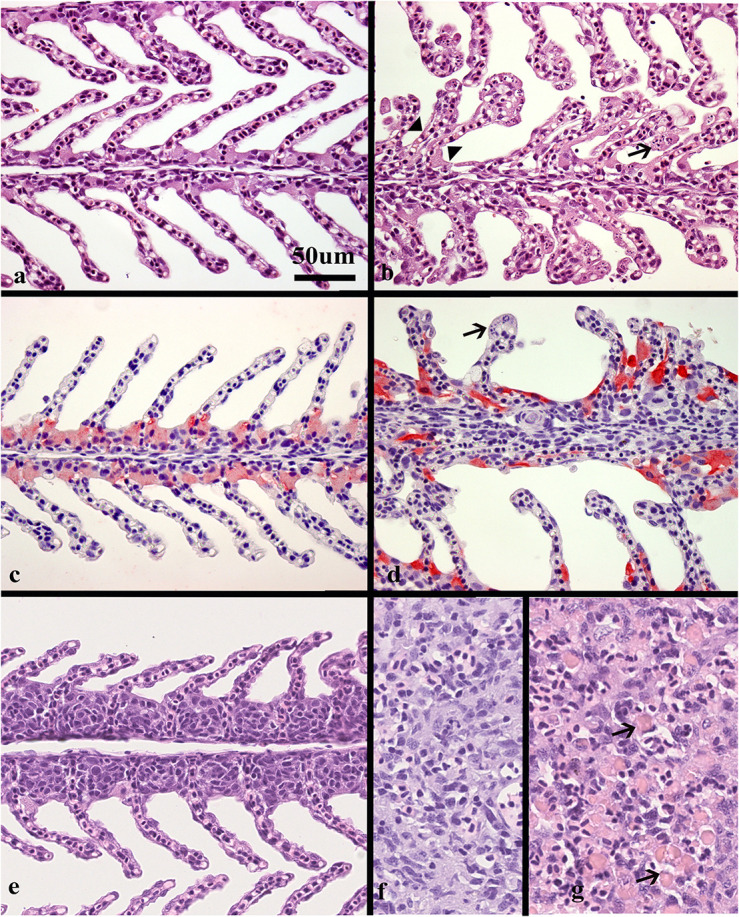
Histological sections of gills and spleen from healthy and SGPV infected fish. **(a)** Sections of healthy gills with normal, thin lamellae stained with hematoxillin and eosin (HE), **(b)** gill from fish sampled from tank I at the start of the outbreak (M-I), gill epithelial apoptosis score of 2,5, SGPV Ct 18,36. Thickening of the lamellae due to apoptotic cells marked by arrow. Some of these cells appear to have the same staining properties as chloride cells. Note also some adhered lamellae (arrowheads). **(c)** Normal, thin lamellae, and chloride cells as detected by immunohistochemistry (light red) located in the filament epithelium. **(d)** gill from fish in tank II during the mortality peak (M-II group), gill epithelial apoptosis score of 1,5, SGPV Ct = 18,88. The chloride cells have a very intense staining and elongated shape compared to the healthy controls. The apoptotic cells (arrow) do not stain. **(e)** Gill epithelial hyperplasia (score 1), from fish in the L-I group with SGPV ct value of 27,9. **(f)** Normal spleen from the C-III group **(g)** spleen from the M-I group showing extensive hemophagocytosis (arrows).

Normal, thin lamellae were seen in the E-III and C-III group (Figures 2a,c). In the M-I group, taken upon the first notice of clinical disease and 2 days before the mortality peak in this tank (Figure 1A), showed extensive apoptosis of gill epithelial cells in all 14 fish (Figure 2b), and five fish with additional sparse (score 0,5) proliferation of the gill epithelial cells. Chloride cells were dislocated, hypertrophic and often elongated, sometimes seen under apoptotic cells along the lamellae (Figure 2d). Further, moderate to extensive hemophagocytosis was seen in hematopoietic tissues of all fish in this group (Figure 2g). PCR confirmed SGPV present in the gills of all fish with a median ct value of 17.2 (range 15.2–18.4).

The L-I group, taken after recovery, and 8 days later in the same tank, showed sparse (score 0,5–1) gill epithelial apoptosis in five out of eight fish and five fish with sparse (score 0,5–1) hyperplasia of the gill epithelial cells (Figure 2e). Sparse to moderate hemophagocytosis were found in hematopoietic tissues of all fish. SGPV was confirmed by PCR in gills of all fish with a median ct value of 24.4 (range 22.2–30.1, Figure 1B).

The M-II group, taken at the mortality peak 2 days after the onset of disease in this tank, showed gill epithelial apoptosis score of 1.5–2 in all eight fish, and hyperplasia of gill epithelial cells of 0.5 in seven fish. As in the M-I group, chloride cells were dislocated, hypertrophic and often elongated, sometimes seen under apoptotic cells along the lamellae. Further, moderate to extensive hemophagocytosis was seen in hematopoietic organs of all fish in this group (Figures 2f,g). Presence of SGPV was confirmed in the gills of all fish with a median PCR ct value of 18.9 (17.5–20.2).

No histopathological lesions were found in the gills or hematopoietic organs in fish from the C-III group or in the SGPV-positive E- III group sampled 11 days later. C-III group was confirmed negative for SGPV by PCR for nine out of ten fish, with one positive (Ct 32.5) and the E-III group sampled 11 days later had a median Ct level of 31.5 (range 29.5–34.5). Some of these results are summarized in Table 1.

### Transcriptomic Analyses

**FIGURE 3:**
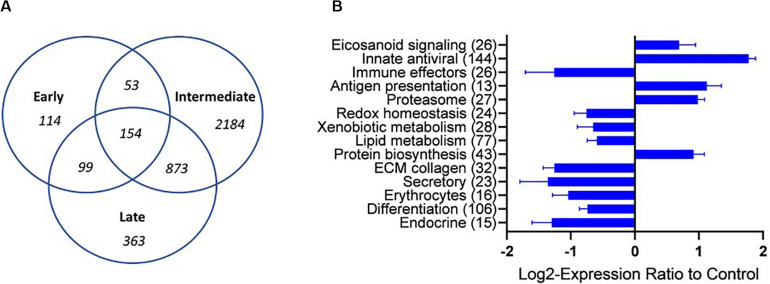
Overview of the transcriptional regulation **(A)** A VENN diagram presenting the number of shared and specific genes between the sampling groups. **(B)** Functional groups of genes (custom vocabulary-STARS) with co-ordinated expression changes at the intermediate stage of infection with SGPV. The numbers of differentially expressed genes are indicated. Data are mean log2-ER ± SE. Differences from control are significant.

Transcriptomic analyses were performed on four representative samples from each of the sample groups (M-I, L-I, M-II, C-III, and E-III). Details of SGPV ct values and histopathological scores of the individuals selected for transcriptomic are given in Table 2. Uninfected C-III samples were used as reference, and up-and downregulation relative to C-III samples were registered (Total transcriptome data in Supplementary File S1). Samples taken during the acute phase of the outbreaks in tank I and II (M-I, M-II) had the most pronounced changes in the transcriptome and showed corresponding transcriptomic responses, strongly indicating that the observed transcriptome response was associated with SGPV infection and SGPVD pathology. The numbers of DEG increased from 420 genes at early phase to 3264 genes at the acute intermediate phase and decreased to 1489 genes from the late sampling. Significant enrichment was observed in functional categories of GO and pathways of KEGG indicating strong immune responses and profound changes at the cellular and systemic level (Supplementary File S2). Several functional groups showed co-ordinated expression changes at the intermediate acute disease phase (Figure 3). Induction was observed in a large group of genes of innate antiviral responses (144 DEG), genes involved in eicosanoid metabolism (lipid mediators of inflammation) and antigen presentation. In contrast, many immune effectors were down-regulated. Suppression of responses to oxidative stress, metabolism of lipids, and xenobiotics was in parallel with up-regulation of protein biosynthesis, which might be exploited by the pathogen. The impact of infection on gill tissue structure and functions was reflected in down regulation of genes encoding regulators of differentiation, endocrine factors, collagens, and secretory proteins. Decreased abundance of transcripts for globins and markers of erythrocytes indicated reduced circulation of blood in the infected tissue.

**TABLE 2:** Detailed information on the samples included in the microarray analyses.

Group ID	Fish	SGPV Ct	Gill apoptosis score	Gill hyperplasia score	Spleen hemophago- cytosis
M-I	1	17,7	2,5	0	1,5
	2	16,5	2,5	0	1
	3	16,6	2,5	0,5	2
	4	16,5	2,5	0,5	2
L-I	1	22,9	0,5	0,5	0,5
	2	22,2	1	0,5	0,5
	3	22,7	0,5	0,5	0,5
	4	22,8	1	0,5	0,5
M-II	1	17,8	2	0,5	2
	2	18,4	2	0,5	2
	3	18,9	1,5	0,5	1
	4	18,2	2	0,5	2
C-III	1	No Ct	0	0	0
	2	No Ct	0	0	0
	3	No Ct	0	0	0
	4	32,5	0	0	0
E-III	1	30,8	0	0	0
	2	29,5	0	0	0
	3	30,2	0	0	0
	4	30,5	0	0	0

*M-I and M-II indicate the peak mortality phase (Tank I and II, respectively), L-I represent fish that have recovered from the diseas in tank I, C-III indicates control samples, and E-III represent early pre-outbreak samples from tank III.*

### Gene Expression Involved in Immune Responses

**FIGURE 4:**
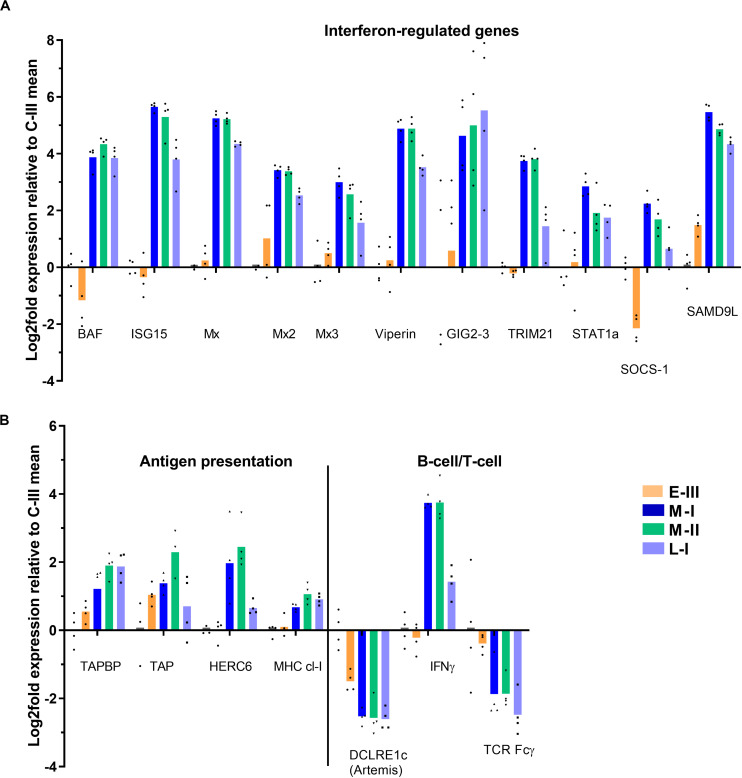
Gene expression reflecting immune responses in gills from Atlantic salmon with SGPVD. Microarray data shown as log2 relative expression relative to control (C-III) mean expression. Mean values (bars) and individual values (dots) are shown. Bar colors: Orange (E-III, early infection), dark blue (M-I, peak infection/clinical disease), green (M-II, peak infection/clinical disease), and light blue (L-I, late infection/resolving disease). **(A)** Selected interferon-stimulated genes. BAF; Barrier of autointegration factor, ISG15; interferon stimulated gene 15, Mx; Myxovirus resistance genes, TRIM; Tripartite Motif protein, STAT; signal transducer and activator of transcription, SOCS; Suppressor of cytokine signaling, and SAMD9L; sterile alpha motif domain-containing 9-like protein. **(B)** Genes involved in antigen presentation and lymphocyte function. TAP; Transporter associated with Antigen Processing; TAPBP; TAP-Binding Protein, HERC; Homologous to E6-AP carboxyl-terminus (HECT)- and Regulator of Chromosome Condensation-1 protein (RCC)-domain containing protein, MHC; multihistocompatibility complex, DCLRE1c; DNA cross-link repair 1C/”Artemis”, IFN; Interferon, and TCR; T-cell receptor.

Salmon gill poxvirus infection triggered a strong typical antiviral response during the clinical acute phase (M-I and M-II), including induction of Mx1-3, RTP3, BAF, Viperin, ISG15, TRIM21, and STAT1a, which lasted partly throughout the regenerating phase (L-I; Figure 4A). The antiviral gene Gig2-3 was especially highly expressed in the late phase, while suppressor of cytokine signaling 1 (SOCS-1) was down-regulated in the early phase of infection (E-III) followed by induction in the acute phase. Genes involved in antigen presentation (TAP, TAPBP, HERC6, MHC class-I, and multiple proteasome components) were up-regulated in the acute disease phase and to lesser extent in the late regenerating phase (Figure 4B). There was no evidence for active adaptive immune responses though several genes were differentially expressed. IFNγ transcripts were increased. The transcripts for lymphocyte associated artemis-like protein, encoded by the DNA cross-link repair 1C gene (DCLRE1c), known to be involved in V(D)J recombination for both B cell antibody genes and T cell receptor genes (20), was downregulated during infection, and the T-cell receptor (TCR) Fcγ gene decreased (Figure 4B).

### Gene Expression Associated With Pathological Findings

**FIGURE 5:**
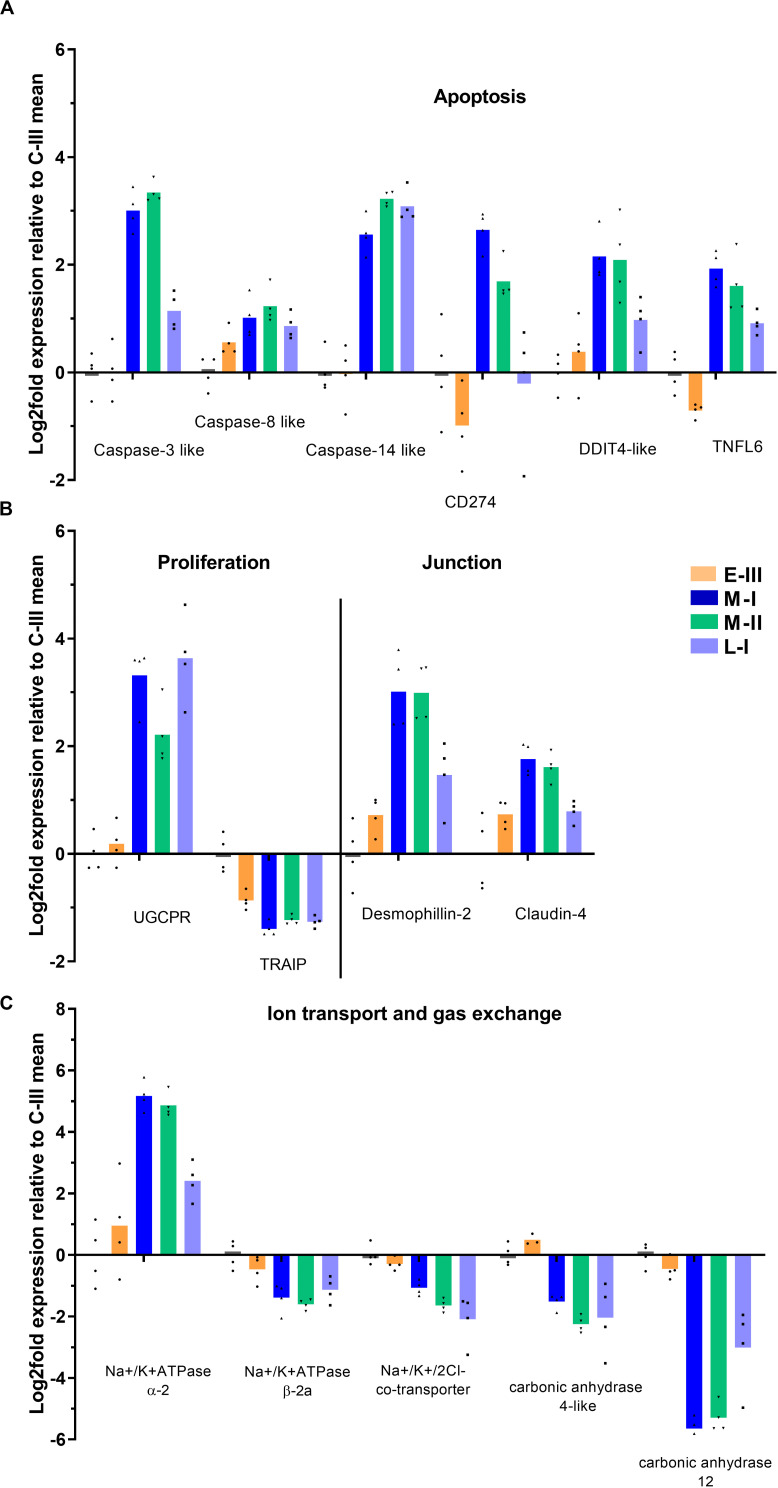
Gene expression reflecting pathological and functional changes in gills from Atlantic salmon with SGPVD. Microarray data shown as log2 relative expression relative to control (C-III) mean expression. Mean values (bars) and individual values (dots) are shown. Bar colors: Orange (E-III), dark blue (M-I), green (M-II), and light blue (L-I). **(A)** Selected genes associated with apoptosis: CD; Cluster of Differentiation, DDIT; DNA Damage-Inducible Transcript, and TNFL; Tumor necrosis Factor Ligand **(B)** Proliferation and junction proteins; UGCPR; upregulator of cell proliferation, and TRAIP; TRAF-interacting protein. **(C)** Genes involved in gill ion transport and gas exchange.

The clinical signs of disease and pathology during the acute and late phase of SGPVD were clearly reflected in the transcriptome (Figure 5A). Induction of pro-apoptotic gene expression (Caspases 3, 8, and 14, CD274, TNFL6) was evident in the acute phase, and Caspase-14 expression was induced also in the late regenerating phase of SGPVD. The proliferating response was associated with induced expression of Upregulator of cell proliferation (UGCPR) lasting into the regenerating phase, and downregulation of the TRAF-interacting protein (TRAIP), a controller of proliferation. TRAIP expression was suppressed already in the early phase of infection (Figure 5B).

Cell-cell junction protein genes were in general not responding strongly at the transcriptional level, but desmophillin-2 and claudin-4 were both upregulated. Genes involved in ion balance (Na+/K + ATPases) and gas exchange (carbonic anhydrases) over the gill surface were also regulated (Figure 5C), which could be associated with the morphological changes observed in chloride cells.

### Gene Expression Involved in Cell Communication and Migration

**FIGURE 6:**
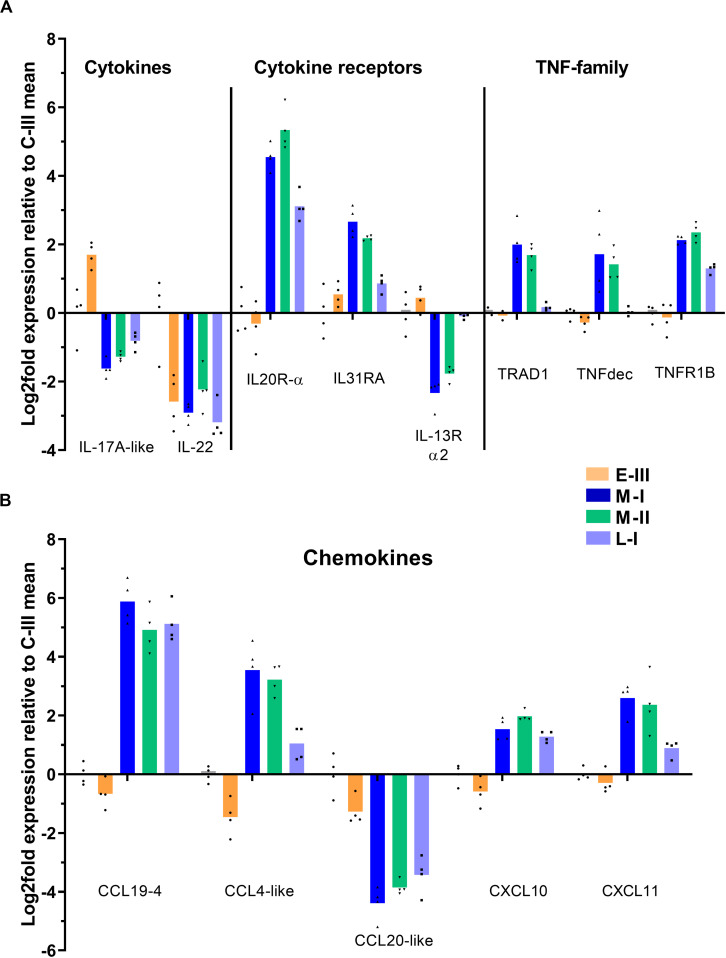
Gene expression reflecting cytokine and chemokine responses in gills from Atlantic salmon with SGPVD. Microarray data shown as log2 relative expression relative to control (C-III) mean expression. Mean values (bars) and individual values (dots) are shown. Bar colors: Orange (E-III), dark blue (M-I), green (M-II), and light blue (L-I). Selected genes associated with cytokines **(A)**: IL; Interleukin, TRAD; TRAF-type zinc finger domain-containing protein, TNF; Tumor necrosis factor, and TNFR; TNF-receptor. **(B)** Chemokines: CCL; CC-motif containing chemokine ligand, and CXCL; CXC-motif containing chemokine ligand.

The main effect seen on cytokine gene expression, was a notable suppression of IL-17A and IL-22 (Figure 6A). Receptor genes for IL20 and IL-31 were induced, whereas the IL-13 receptor was suppressed (Figure 6A). Several inhibitory members of the tumor necrosis factor (TNF) alpha-family of cytokines were upregulated in the acute phase of disease, including TRAF-type zinc finger domain-containing protein 1 (TRAD1), soluble TNF receptor (TNFR1b), and TNF decoy receptor (Figure 6A).

The regulation pattern of chemokines showed an opposite regulation of CCL19-4 (strongly upregulated) and CCL20 (strongly downregulated) starting in the acute phase and lasting through the regenerating phase of SGPVD (Figure 6B). Induction of CCL-4/macrophage inflammatory protein-1β, CXCL10, and CXCL11 transcripts were also seen in the peak phase of disease (Figure 6B).

### Gene Expression Related to Mucosal Protection

**FIGURE 7:**
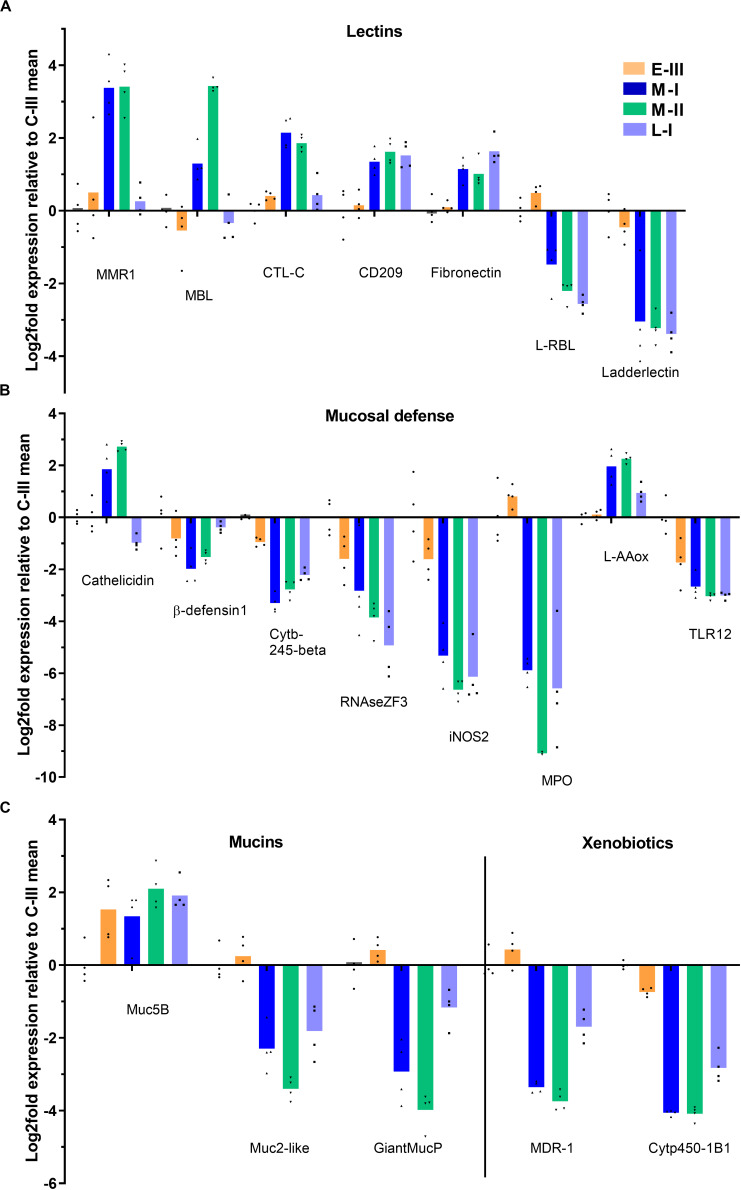
Gene expression reflecting mucosal defense in gills from Atlantic salmon with SGPVD. Microarray data shown as log2 relative expression relative to control (C-III) mean expression. Mean values (bars) and individual values (dots) are shown. Bar colors: Orange (E-III), dark blue (M-I), green (M-II), and light blue (L-I). **(A)** Selected genes associated with lectins: MMR; Macrophage mannose receptor, MBL; Mannose –binding lectin, CTL; C-Type lectin **(B)** Mucosal protection: iNOS; inducible NO-Synthase, MPO; Myeloperoxidase, L-AAox; L-Amino-acid oxidase, and TLR; Toll-like receptor. **(C)** Mucins and xenobiotic.enzymes: Muc; mucin, MDR; Multiple Drug-Resistance protein, and Cytp; cytochrome protein.

Several genes involved in mucosal protection were affected during the disease course. Lectins, important in biological recognition of cells and proteins and for binding/blocking of infectious agents, showed different regulation patterns (Figure 7A). Mannose binding lectin, macrophage mannose receptor 1 (MMR1), and C-type lectin (CTL) specifically increased in the acute phase of SGPVD. The CD209 receptor and fibronectin were upregulated into the late regenerating phase, and rhamnose-binding lectin and Ladderlectin were downregulated in the acute and regenerating phase (Figure 7A). Several genes involved in enzymatic bactericidal responses were suppressed into the late regenerating phase of disease (L-I; Figure 7B), including the bactericidal RNAse ZF3, myeloperoxidase (MPO), and cytochrome b-245β (Cytb-245), along with iNOS2, Toll-like receptor 12 (TLR12), and β-defensin. In contrast, cathelicidin and the bactericidal L-amino acid oxidase (L-AAox) were upregulated in the acute phase (Figure 7B). Mucin genes were also differentially affected (Figure 7C), and whereas the Mucin 5B gene was induced, Mucin-2-like and Giant mucin protein genes were suppressed. The xenobiotic proteins Cytochrome p450 1B1-like (Cyt450-1B1) and multidrug recistance protein 1-like (MDR-1) were also downregulated in the regenerating phase (Figure 7C).

## Discussion

In this study we investigated aspects of a field outbreak of SGPVD. We sampled gills from Atlantic salmon presmolts and covered the early phase of infection, the acute mortality phase, the resolving phase of the disease and control fish from the same group and facility. Virus levels, gill pathology typical for SGPVD and transcriptional antiviral responses all closely associated with the acute clinical phase of the disease and is in accordance with previous studies (3, 8), and again shows that this gill disease is caused by SGPV.

In all three tanks sampled in the outbreak a peak mortality phase lasting for 4 days was seen. The reason for this strict time frame cannot be elucidated from the limited number of samples taken from this outbreak, but would be an interesting focus for subsequent studies. The mortality was highest in the first tank affected by the outbreak, then decreased in the tanks affected later. This decrease in mortality in tank I, II, and III, respectively, was not reflected in any of the analyses, and not in the transcriptome. It should be noted, however, that fish were sampled from tank I at the day of the onset of the outbreak, whereas fish from tank II were sampled during peak mortality.

Peak virus levels were clearly associated with gill epithelial apoptosis and chloride cell pathology, whereas lower virus levels seen in the recovery phase were associated with epithelial hyperplasia. Both hyperplasia and increased life span of the cells may thicken the gill epithelium. This response reduces uptake of harmful substances and saves oxygen and energy expensive transport. However, a reduced gill surface area may impair the systemic respiration in a critical way: limiting carbon dioxide excretion initially, then, if sufficiently severe, compromise oxygen uptake (21). In this study, however, the degree of epithelial hyperplasia was only sparse and not associated with clinical signs or mortality in the late phase of disease. In the context of SGPVD, the drivers of this hyperplasia are unknown. It could be a protective response directed by the host, but it could also be initiated by the virus itself as a way of generating new susceptible host cells, as seen in other poxviruses (22). The similarity between observations here and previous reports on SGPVD were very clear (3, 8).

The innate antiviral response observed in transcriptome data from the infected gills is strong at the peak of infection. Innate antiviral responses have been extensively studied in salmonid fish [reviewed in (23, 24)]. Transcriptome analyses of Atlantic salmon tissues and cells infected with virus and treated with poly(I:C) has revealed more than hundred virus responsive genes – VRG (19). Until recent, antiviral responses of salmonid fish have been investigated in association with RNA viruses and double-stranded RNA applied as surrogate infection. Recently, strong induction of this group was reported in skeletal muscle of Atlantic salmon injected with bacterial DNA in a form of plasmid (25, 26). SGPV is the first systematically explored DNA virus of Atlantic salmon and it is interesting to observe that it induces a similar innate antiviral response. VRG showing the highest responses to RNA viruses (ISG15, Mx, RTP1-3, Viperin, IFIT5, Vlig, and Gig2-3) were the most upregulated in this study. A gene encoding for a macrodomain-containing protein predicted to inhibit interferon induction is previously described in SGPV (3), and in other poxviruses like the Vaccinia virus, blocking of IFN signaling is reported as one of the counterattack mechanisms on the antiviral response (27). Still, signs of interferon inhibition was not observed in SGPV-infected gills in our study, as many interferon-induced genes were strongly upregulated. Some of these genes encode proteins shown to directly affect the replication of other poxviruses, including ISG15 (28), Barrier to Autointegration Factor/BAF (29), sterile alpha motif domain-containing 9-like (SAMD9L) (30), and suppressor of cytokine signaling (SOCS)-1 (31). Although upregulated at the transcriptional level, the antiviral gene products may be inhibited post-translationally by SGPV host interaction proteins, as shown for other poxviruses (27, 29, 30).

The transcriptome profile in the SGPV-infected gills lacks signs of a proinflammatory cytokine response. Instead, there is an expression of several inhibitory TNF-family proteins, including TNF decoy protein, soluble TNF-receptor and the TRAF inhibitor TRAD1, that may stop the inflammatory response triggered by TNFα. In addition, proinflammatory signaling can be inhibited by SOCS-1, which is also reported to serve a role in regulating T-cell differentiation and in particular inhibit the Th17 cells that produce IL-17 (32). Interestingly, we see a reciprocal regulation of the SOCS-1 gene (down) and the IL-17A gene (up) during early phase of SGPV infection, prior to the IFN-regulated induction of SOCS-1 in the clinical phase, when also the IL-17A gene regulation turns. SOCS-1 is shown to directly inhibit poxvirus replication, and a SOCS-1 derived peptide can function as an antiviral treatment against lethal poxvirus infection (31). This increases the likelihood that SGPV may inhibit SOCS-1 expression as a counteractive mechanisms in the early phase of infection, and this hypothesis will be subject to our further study.

We see an upregulation of several genes involved in antigen presentation, but adaptive immune responses are not observed within the time frame of this trial. Levels of IgT and IgM, and also of standard T-cell markers like CD4 and CD8, are low and not increased during or after the peak of infection, as would be expected if a proliferation was induced. The low inflammatory cytokine response and altered chemokine responses may have recruited less immune cells to the infection site, but the time-frame of this study may also be to short to conclude that this is due to a general suppression of adaptive immunity in the gills, as the “late” sampling point is just 1 week after the peak of infection. In other Atlantic salmon viral diseases we have seen that specific antibodies were not produced until 2 weeks after the infection peak (33).

A main characteristic of SGPVD is the extensive gill epithelial apoptosis in the acute phase. The transcriptome data revealed increased gene expression of several caspase isoforms associated with apoptosis during this phase. Caspases 3 and 14 were the strongest responding caspase genes in gills from this SGPVD outbreak, followed by caspase 8. Caspase 8 is known to be responsible for activating caspase 3, and their functions are therefore linked (34). Caspase 3 activity is often seen in gills of different fish species as a response to non-infectious stressors, like hypoxia or high salinity (35). TNFSF6, or FAS-ligand/CD95-ligand, is a TNF-family member that can be involved in initiating suicidal activation-induced cell death (AICD) via the TNFRSF6/CD95/FAS receptor (36). This system has previously been implicated in mediating alveolar epithelial cell injury in mammals (37). The TNFSF6-induced mechanism involve activation of both caspase 8 and caspase 3, and the coordinated activation of these three genes indicate that the pathway may play a role in SGPV-mediated cell death.

We also see upregulation of CD274, also named death-ligand 1 (PD-L1), which is best known for its role in suppressing lymphocyte proliferation, activation and adaptive immunity (38). Thus, CD274 could be associated with immunosuppression in SGPVD.

Dying epithelial cells are themselves stimulators of proliferation. An experiment performed in zebrafish demonstrated that basal stem cells can engulf apoptotic bodies and thereby induce proliferation in a caspase 3-dependent manner (39). The zebrafish experiment was performed in skin keratinocytes, but is likely to be relevant for the gill epithelium as well. URGCP (upregulator of cell proliferation, also known as URG4) is previously shown to be upregulated following virus infection, resulting in cell growth/proliferation (40), and the gene may play a role in proliferation of the gill epithelium also in SGPV-infected salmon. TRAIP is a controller of proliferation, and TRAIP knock-out mice die from aberrant proliferation and apoptosis (41). TRAIP gene expression was suppressed in samples from early SGPV infection (E-III), when no gill pathology was seen, and its regulation could potentially initiate some of the pathological effects.

The regulation of several genes clearly associated with SGPVD pathology suggest that a set of transcriptonal biomarkers of pathology could be developed. Transcripts from gill biopsies have previously been used to examine infection biomarkers in wild salmonids (42). Such biopsies can potentially be taken from live fish and analyzed instead of, or as a supplement to, histopathological examination, and provide information on the state of disease. The transcriptome may also indicate how the virus affects gill function and interacts with host defense, and thereby have predictive value regarding future disease outcome. Most likely, no single biomarkers will be able to predict the outcome of SGPV-infection, but a profile of several selected biomarkers may.

Chloride cells in the gills are important to maintain the ion balance in the fish. In this study, the chloride cells were dislocated, hypertrophic and with an unusual shape in the acute phase of SGPVD. The transcriptome analysis showed increased Na+/K+ −ATPase protein expression, and altered gene regulation related to the chloride cell functions. Na+/K+ −ATPase isoform genes (α-2 vs β-2a) are differentially regulated in the acute phase of SGPVD. Na+/K+ −ATPase isoform exchange is a well established marker of smoltification, but this is linked to other isoforms (α-1a vs β-1b) (43). We also observe a notable suppression of genes encoding two carbonic anhydrases, which are zinc metalloenzymes essential for CO_2_ exchange in gills. Carbonic anhydrase subtype 4 catalyzes CO_2_ exchange from plasma in the gills of several fish species (44). Although carbonic anhydrase subtype 12 is less studied in fish, mutations in its human counterpart has been linked to cystic fibrosis (45), which indicate a potentially important role for respiratory function. It is likely that downregulation of the carbonic anhydrases could affect respiration and add to the clinical effects of SGPV infection.

Mucus secretion protects fish from environmental stressors and infection. Among the mucin genes regulated here, the secreted mucin Muc5 is upregulated in contrast to a downregulation of other mucins. The same observations were made during the course of amoebic gill disease (46). Upregulation of mucin 5B was seen already prior to the clinical phase in the absence of pathological findings. An upregulation of the Th2- type cytokines IL-4/IL-13 was also reported during the course of AGD in the same trial (46), but this was not seen in our study.

Other cytokine genes, however, show interesting regulation in response to SGPV-infection. IL-22, a cytokine known to induce inflammation and promote wound closure and recovery after epithelial damage, is strongly suppressed during SGPV infection. Interestingly, the suppression is observed during early infection, suggesting that this could be a virus-regulated effect. This inhibitory effect contrasts previous findings in rainbow trout gills after infection with *A. salmonicidae*, where IL-22 was strongly upregulated (47). In that study, the infection was shown to increase the number of IL-22 producing cells in the gill epithelium and interbranchial lymphoid tissue (47). IL-22 is expressed by innate T-cells (γδ T cells), abundant in mucosal tissue (48). In line with IL-22 suppression, we also see lower expression of the gene encoding the Fcγ subunit of the γδ TCR, which could indicate loss of innate T-cells in the gills. IL-22 is an important regulator of mucosal defense in general (49), and regulates β-defensin and several antibacterial effector proteins in the mucosa (50), which we also see suppressed in our data. In addition, IL-22 also regulates proliferation of the epithelium and tissue repair (48). Hence, many of the effects reflected in the current transcriptome and also during the disease course could potentially be caused by suppressed expression of IL-22.

Innate γδT cells carry the CCR6-receptor and are attracted to tissue mainly by the chemokine CCL20 (51). In SGPV-infected gills we see a coordinated suppression of CCL20 and IL-22 in the early phase of infection (E-III), and the γδ T-cell marker TCR Fcγ, is also suppressed during the peak of infection. The cytokine/chemokine suppression found during early SGPV infection, could indicate the involvement of a virus-encoded protein in the regulation. In contrast to CCL20 suppression, CCL19 is strongly induced in SGPVD gills. CCL19 is involved in T-cell recruitment during viral infection, and binds to the CCR7 receptor which is expressed on immune cells and cancer cells (52). CCL19 is also reported to promote AICD of T-cells, an effect associated with increased expression of FAS ligand (FASL/TNFSF6) (53). The role of SGPV in controlling changes in cytokine and chemokine responses to promote its own replication and dissemination is unknown, but other poxviruses are known to interfer with host cytokine and chemokine signaling through both intracellular and extracellular mechanisms (27).

The genes regulated in the early phase of infection represent potential early biomarkers of SGPVD. Since impairment of IL-22 is likely to be associated with suppression of several proteins involved in mucosal defense, it is not unlikely that this may pave the ground for secondary, opportunistic infections and increased sensitivity to environmental stressors in the aftermath of a SGPV-infection (6). This will be addressed in future experimental trials.

This study strongly indicates that SGPV infection results in disturbed mucosal defense and tissue regeneration in surviving fish that can be reflected in the transcriptome, most likely linked to the cytokine/chemokine environment and aberrant T-cell recruitment. Taken together, the findings are in line with the previously described clinical and pathological observations in SGPVD and also strengthen the hypothesis that SGPV infection can pave the way for secondary pathogens. Not only because the physical barrier can be disrupted, but also from an immunological perspective.

## Data Availability Statement

The microarray data have been uploaded to GEO (GSE151463).

## Ethics Statement

Animals were sampled in relation to a disease in aquaculture and not subject to experimental procedures. Sampled animals were immediately killed by an anesthesia overdose. Written informed consent was obtained from the owners for the participation of their animals in this study.

## Author Contributions

MKD, MCG, OBD, and AK designed the study. SB sampled fish and gathered clinical data.MCG performed histological analyses. AK, GT, and SA performed and analyzed microarray data. MKD, MCG, and AK were responsible for data interpretation, figures, and wrote the manuscript. OBD contributed to the discussion of the manuscript. All authors read the final manuscript and approved submission.

## Conflict of Interest

SB was employed by MOWI ASA. The remaining authors declare that the research was conducted in the absence of any commercial or financial relationships that could be construed as a potential conflict of interest.
